# Expression of Septin4 in *Schistosoma japonicum*-infected mouse livers after praziquantel treatment

**DOI:** 10.1186/s13071-015-0640-9

**Published:** 2015-01-13

**Authors:** Dandan Zhu, Ke Song, Jinling Chen, Jianxin Wang, Xiaolei Sun, Hongyan Qian, Xijuan Gu, Lingbo Zhang, Yongwei Qin, Yinong Duan

**Affiliations:** Department of Pathogen Biology, School of Medicine, Nantong University, 19 Qixiu Road, Nantong, 226001 Jiangsu People’s Republic of China; Laboratory Medicine Center, Affiliated Hospital of Nantong University, 20 Xisi Road, Nantong, 226001 Jiangsu People’s Republic of China; Cancer Research Center, Affiliated Tumor Hospital of Nantong University, Nantong, 226001 Jiangsu People’s Republic of China; Nantong University Xinglin College, Nantong, 226001 Jiangsu People’s Republic of China

**Keywords:** Schistosoma japonicum, SEPT4, Inflammation, Liver fibrosis

## Abstract

**Background:**

Septin4 (SEPT4) exists widely in human tissues and is related to mechanical stability, actin dynamics, membrane trafficking, viral replication and apoptosis. Data from many studies have suggested that SEPT4 plays a significant role in liver fibrosis. SEPT4 is down-regulated in the model of CCl_4_ and BDL treated liver fibrosis. However, it is up-regulated and peaked at 12 weeks post-infection (p.i.), and then decreased subsequently in *Schistosoma japonicum* (*S. japonicum*) egg-induced liver fibrosis. The aim of this study was to observe the dynamic alteration of SEPT4 after the treatment of praziquantel (PZQ) in ICR mice infected with *S. japonicum*.

**Methods:**

Expression of SEPT4 was determined by western blot, immunofluorescence and qRT-PCR. And pro-inflammatory cytokines IL-6 and TNF-α were detected by qRT-PCR. The number of eggs, the diameter of egg granulomas and fibrosis-associated genes were also measured.

**Results:**

Our results showed that the granulomatous inflammation was reduced, whereafter the expression of SEPT4 on hepatic stellate cells (HSCs) was decreased after PZQ anti-schistosome therapy. And the variation tendency of SEPT4 had positive correlation with the inflammatory response in the area of *S. japonicum* egg granulomas.

**Conclusions:**

Based on these findings, the inhibition of the expression of the SEPT4 by PZQ might be due to alleviation of the inflammatory response at the chronic and advanced stage of *S. japonicum* infection.

## Background

Schistosomiasis japonica, one of the three major human schistosomiases, is still a public health issue in the People’s Repubic of China [1]. All evidence indicates that morbidity of the disease is caused by *Schistosoma japonicum* (*S. japonicum*) eggs [2]. The eggs secrete soluble egg antigens (SEA) and elicit an egg-induced granulomatous response, leading to portal hypertension and liver fibrosis [3-5].

It is well known that hepatic stellate cells (HSCs) are located in the space of Disse of the liver sinusoid and store vitamin A in normal liver [6,7]. Following chronic injury to the liver, HSCs change from a quiescent form to an activated phenotype so that they lose the ability to store vitamin A, begin to express α-smooth muscle actin (α-SMA) and produce extracellular matrix (ECM) [2,8,9]. It has been identified that activated HSCs are shown to be present in the periphery of egg granulomas in murine and human *S. japonicum* infection [10]. Thus, HSCs play a pivotal role in *S. japonicum-*induced liver fibrosis [11].

As a family of GTP-binding proteins, Septins are widely found in eukaryotes. They are considered as an essential component of the cytoskeleton [12], and have multiple cell functions such as cell division, polarity and membrane remodeling [13]. Septin4 (SEPT4), belongs to a subset of Septins, is widely expressed in human tissues including brain, heart, liver, lymphocyte and testes. It participates in many important physiological processes such as membrane trafficking, viral replication and apoptosis [14]. Some researches have already demonstrated that SEPT4 is expressed in HSCs in the model of carbon tetrachloride (CCl_4_) and bile duct ligation (BDL)- treated liver fibrosis and plays an essential role during liver fibrosis [15-17]. Our previous researches have indicated that SEPT4 is up-regulated in the activated HSCs induced by lipopolysaccharides (LPS) *in vitro* [18], and *in vivo* it increases gradually until 12 weeks post-infection (p.i.) and decreases subsequently in *S. japonicum* egg-induced liver fibrosis [19]. However, SEPT4 is down-regulated in the model of CCl_4_ and BDL treatment liver fibrosis [15,16].

During the process of *S. japonicum* egg-induced liver fibrosis, the major pathologic damage is the granulomatous inflammation that occurs around eggs trapped in the liver [20]. IL-6 recruits numerous cell types, including macrophages, fibroblasts, eosinophils and mast cells, to the sites of acute hepatic inflammation [21]. Moreover, TNF-α is required for the granuloma formation with local collagen secretion in SCID mice [22]. Administration of recombinant murine TNF-α to mice with chronic schistosomal infection has increased the size of liver granulomas, and injections of polyclonal anti-TNF-α into acutely infected mice have suppressed the size of developing granulomas [23]. Given that the pro-inflammatory cytokines are closely associated with schistosome egg granuloma formation in the acute stage of schistosome egg-induced liver fibrosis [24], and the granulomatous inflammatory process induces activation of HSCs [25], we wonder if the inflammation caused by the infection of *S. japonicum* can cause the up-regulation of SEPT4 in the mouse model of *S. japonicum* egg-induced liver fibrosis. As an effective anti-schistosomal drug, praziquantel (PZQ) has been used to treat human schistosomiasis over 30 years [26]. It has various effects on different developmental phases of schistosomes. In human and animal models, PZQ can eliminate the adult worms of *S. japonicum*, but has less effects on egg and schistosomula [27]. After the death of adult worms, the number of excreted schistosome eggs is reduced correspondingly. Additionally, the egg-induced granulomatous inflammation alleviates and the activated HSCs reduce accordingly after the treatment of PZQ. PZQ exhibits a potential anti-inflammatory effect [28,29] and probably down-regulates the expression of pro-inflammatory cytokines, resulting in the decreased expression of SEPT4.

Thus, in this study, we observed the dynamic changes of pro-inflammatory cytokines and SEPT4 in *S. japonicum-*induced liver fibrosis before and after the treatment of PZQ in order to explore the possible factors of leading to the up-regulation of SEPT4 in the chronic and advanced stage of *S. japonicum* egg-induced liver fibrosis.

## Methods

### Animals and drugs

Seventy-two male ICR mice, 18-22 g, were purchased from Center for Experimental Animals of Nantong University (Nantong, China). *S.japonicum* cercariae were obtained from the infected intermediate host snail *Oncomelania hupensis* (Jiangsu institute of parasitic diseases, Wuxi, China). PZQ (Sigma, USA) suspension was dissolved in 1% carboxymethyl cellulose and freshly prepared. Animal care and experiments were approved by the Animal Ethics Committee of Nantong University.

### Model establishment and PZQ treatment

Six mice were used as the normal control group, and the others were infected with 20 ± 2 *S. japonicum* cercariae via shaved skin of abdomen. Thirty-six mice were taken randomly as the liver fibrosis group, every six mice from the liver fibrosis group were sacrificed at 6, 8, 12, 16, 20, 24 weeks p.i.. Retained thirty mice were taken as the PZQ anti-parasite group. All of thirty mice were treated with PZQ suspension (250 mg/kg/24 hours) for 3 days by gastric gavage at 6 weeks p.i., six mice from the PZQ anti-parasite group were sacrificed at 8, 12, 16, 20, 24 weeks p.i..

### Measurement of egg number

To obtain the total number of eggs in the liver, 500 mg liver tissue per mouse was weighted and put into a 50 ml tube with 20 ml of a 10% KOH solution (p/v). The livers were digested at 37°C for 2 h. The solutions were centrifuged at 600 g for 5 min and the eggs were resuspended in 10 ml saline. An average of five counts was obtained per 50 μl solution under an optical microscope to calculate the number of eggs per gram of liver tissue.

### Hematoxylin-eosin staining and Sirius red staining

Liver specimens were fixed in 4% paraformaldehyde in PBS and dehydrated in a graded sucrose series. The cryosections were sliced up at 7 μm thickness with Leica CM1950 Cryostat (Leica,Germany). Then liver cryosections were stained with hematoxylin-eosin (HE) and aqueous saturated solution of picric acid containing 0.1% Sirius Red (Sigma, USA). Five random fields of microscope of egg granuloma were taken in the liver cryosections of each mouse with H&E staining by Leica DM 5000 B microscope (Leica, Germany) and photographs were processed and analyzed on ImageJ analysis software. The calculational method of red-stained collagen fibers with Sirius red staining was followed by the same steps above. The diameters of the five largest granulomas in each cryosection were measured by ImageJ analysis software. Average granuloma diameter calculated for three mice each group.

### Immunofluorescence

The liver cryosections were blocked with serum at room temperature for 1 h before staining. For double staining of desmin and SEPT4, cryosections were labelled with a goat primary antibody against desmin (Santa Cruz, USA) and a rabbit antibody against SEPT4 (Santa Cruz, USA) at 4°C for 16 h, then Alexa Fluor® 568 secondary antibody (Invitrogen, USA) and FITC secondary antibody (Jackson, USA) were added to the cryosections and incubated at room temperature for 1 h. After staining for desmin and SEPT4, the nuclei were counterstained with Hoechst 33342 (Sigma, USA). Images were visualized under Leica DM 5000 B microscope (Leica, Germany) and analyzed with Leica Q Win Plus imaging software (Leica, Germany).

### Western blot

Liver tissues were homogenized in RIPA lysis buffer (Beyotime, China) containing phenylmethanesulfonyl fluoride (PMSF). After quantification, protein samples were boiled in 5 × SDS-PAGE loading buffer for 10 min. Then samples were added in wells of a 10% (w/v) polyacrylamide gel and transferred to a polyvinylidene fluoride (PVDF) membrane. The membranes were blocked with 10% milk at room temperature for 1 h and performed with glyceral-dehyde-3-phosphate dehydrogenase (GAPDH) (Goodhere, China), α-SMA (Santa Cruz, USA), and SEPT4 (Santa Cruz, USA) antibodies at 4°C for 16 h. Then the membranes were incubated with horseradish peroxidase (HRP)-conjugated secondary antibodies at room temperature for 1 h. The membranes were detected using enhanced chemiluminescence (ECL) kit (Merck, Germany).

### Quantitative real-time polymerase chain reaction (qRT-PCR)

Liver tissues were homogenized in Trizol (Invitrogen, USA) in tissue homogenizer and the total RNA was extracted following the manufacturer’s protocol. The reverse transcription reaction used RevertAid™ First Strand cDNA Synthesis Kits (Thermo Fisher Scientific, USA). qRT-PCR was performed using the SYBR®Premix Ex Taq™ RT-PCR Kit (Takara, Japan) in the Eco Real-time PCR system (Illumina, USA).

### Statistical analysis

Prism software (GraphPad) was used to determine the statistical significance of differences in the means of experimental groups. Data of two groups were analyzed for statistical significance with Student’s *t*-test. Multiple comparisons were made by using one-way ANOVA.

## Results

### Egg counts of liver

**Figure 1:**
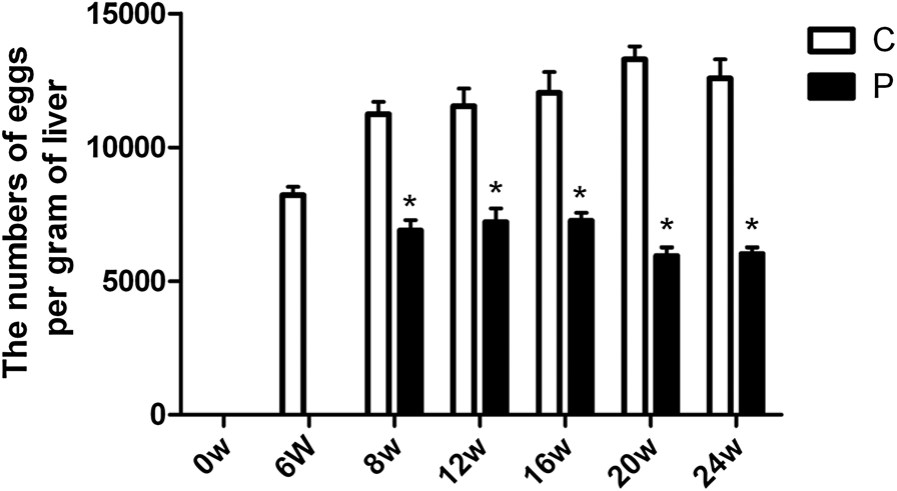
**Liver egg counts.** The numbers of eggs per gram of liver were decreased by the anti-parasite therapy of PZQ. * p < 0.05 vs the control group (without the treatment of PZQ).

In the infected group, the numbers of eggs per gram of liver were dramatically increased, while the numbers were decreased by anti-parasite therapy of PZQ (Figure 1). These results demonstrate that adult worms are killed and the number of excreted schistosome eggs is alleviated after PZQ anti-parasite therapy.

### General histology and change of fibrosis-associated genes

**Figure 2:**
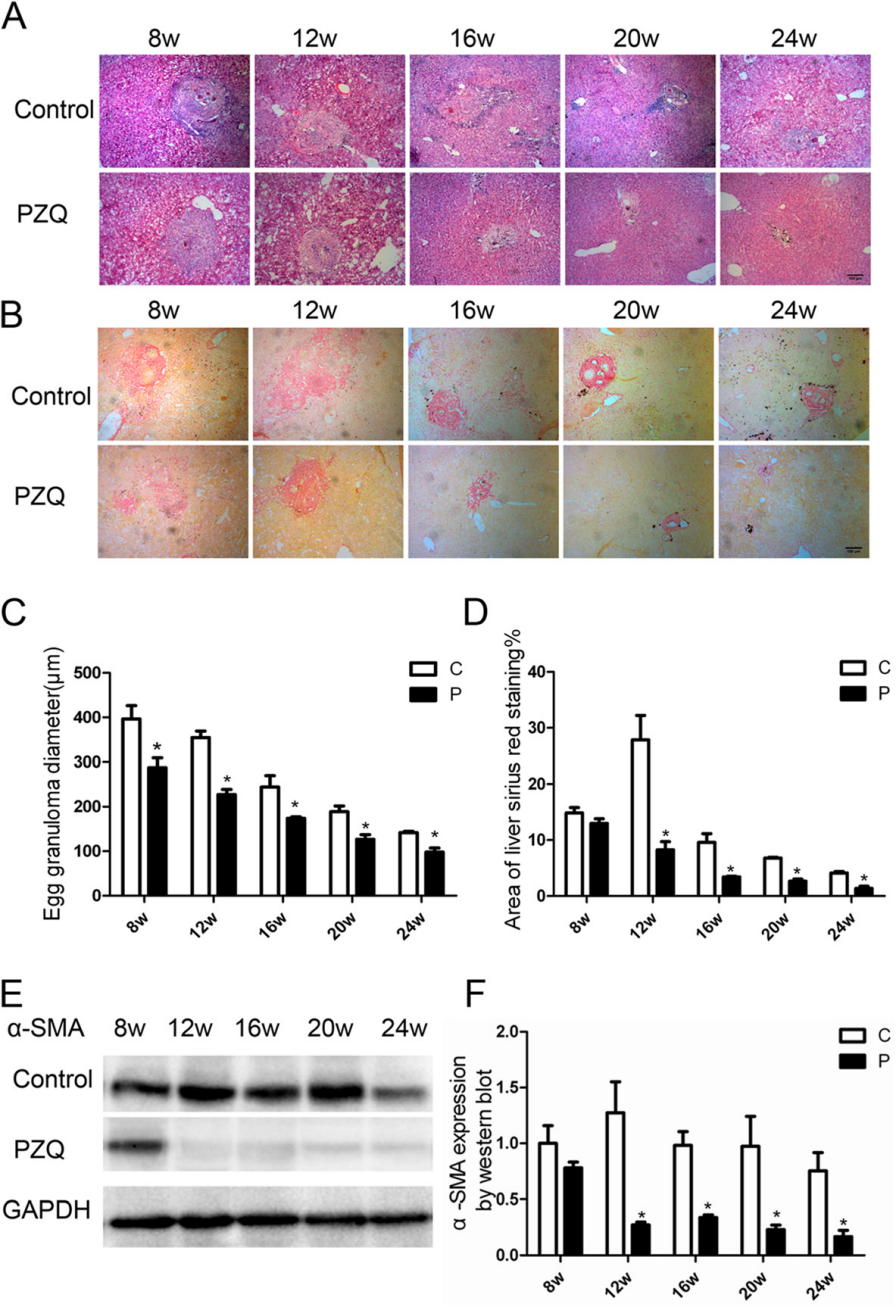
**Change of the diameter of egg granulomas, area of collagen deposition and expression of α-SMA. (A)** Granuloma diameter. **(B)** Area of collagen. **(C-D)** Statistical analysis of images in hematoxylin-eosin staining and sirius red staining by software GraphPad, respectively. **(E)** Expression of α-SMA was reduced after the anti-parasite treatment of PZQ. **(F)** Statistical analysis of the expression of α-SMA by software GraphPad. Bar: 100 micrometer. *p < 0.05 vs the control group(without the treatment of PZQ).

Granuloma induced by the schistosome egg is an inflammatory reaction and the diameter of egg granulomas reflects the degree of inflammatory response. In the infected group, egg granulomas of *S. japonicum* egg-induced liver fibrosis tissues at the acute stage were observed at 6 weeks p.i. and the diameter of egg granulomas were largest at this stage. With the development of pathologic process, the size of egg granulomas at the chronic phase (12 weeks) was smaller than that at the acute phase. Then the diameter further decreased at the advanced phase (24 weeks). After the treatment of PZQ at 6 weeks, the diameters of egg granulomas were significantly decreased in PZQ treatment group compared with infected group during the same time (Figure 2A). Sirius Red staining of liver fibrosis tissues showed the presence of collagen around egg granulomas. In the infected group, along with the infection development, the area of collagen deposition increased gradually and peaked at 12 weeks. Thereafter, the areas reduced significantly at the advanced phase of *S. japonicum* egg-induced liver fibrosis. By contrast, the areas of collagen were down-regulated after PZQ treatment compared with infected group (Figure 2B). As the marker of activated HSCs, α-SMA expressed in the peripheral regions of egg granulomas after post-*S. japonicum* infection [10]. Here, we detected the dynamics of α-SMA by western blot analysis. α-SMA peaked at 12 weeks and down-regulated gradually. However, compared with the infected group, α-SMA peaked at 8 weeks and had a significant decrease between 12 weeks and 24 weeks in PZQ treatment group (Figure 2E, F). These results indicate that the treatment of PZQ could alleviate the inflammation response and the degree of liver fibrosis.

### Dynamics of pro-inflammatory cytokines TNF-α and IL-6 before and after PZQ treatment

**Figure 3:**
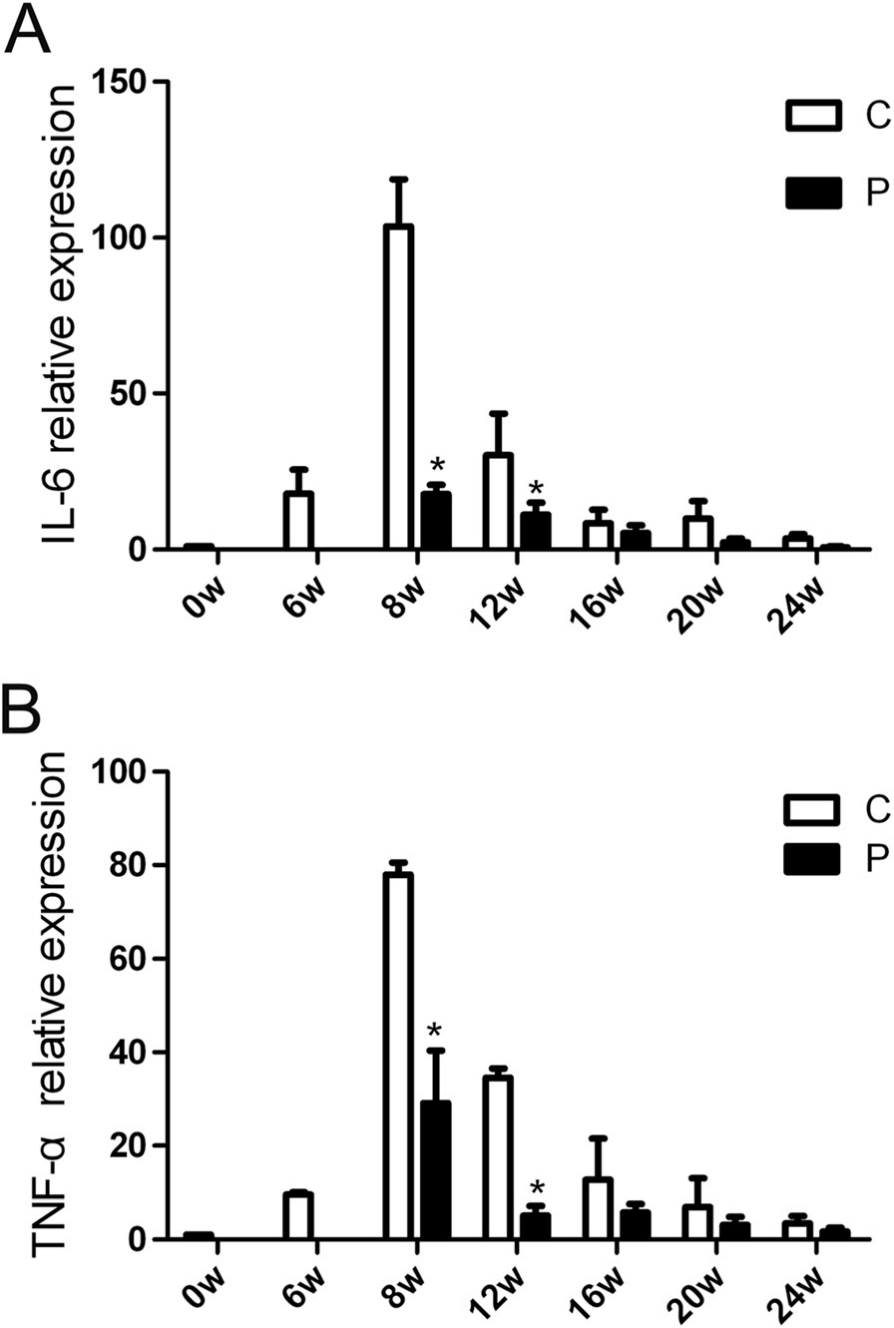
**Gene expression of pro-inflammatory cytokines TNF-α and IL-6 in the livers from the time course study measured by qRT-PCR.** Results are expressed as fold amplification over normal, uninfected liver following normalization with GAPDH. Transcriptional levels of IL-6 **(A)** and TNF-α **(B)** in the liver of the treated mice were significantly reduced compared with the infected mice. * p < 0.05 vs the control group (without the treatment of PZQ).

TNF-α and IL-6 are thought to play significant roles in *S. japonicum* egg induced granuloma formation. Then, we evaluated the changes of TNF-α and IL-6 before and after PZQ treatment in liver tissues. By qRT-PCR, relative expression of TNF-α and IL-6 peaked at 8 weeks p.i. and fell significantly at 12 weeks. Both of them were decreased after anti-parasite treatment of PZQ (Figure 3A,B). These results suggest that anti-parasite treatment of PZQ inhibits transcriptional levels of pro-inflammatory cytokines TNF-α and IL-6 expressions in the liver of schistosomiasis mouse.

### Dynamics of SEPT4 before and after PZQ treatment

**Figure 4:**
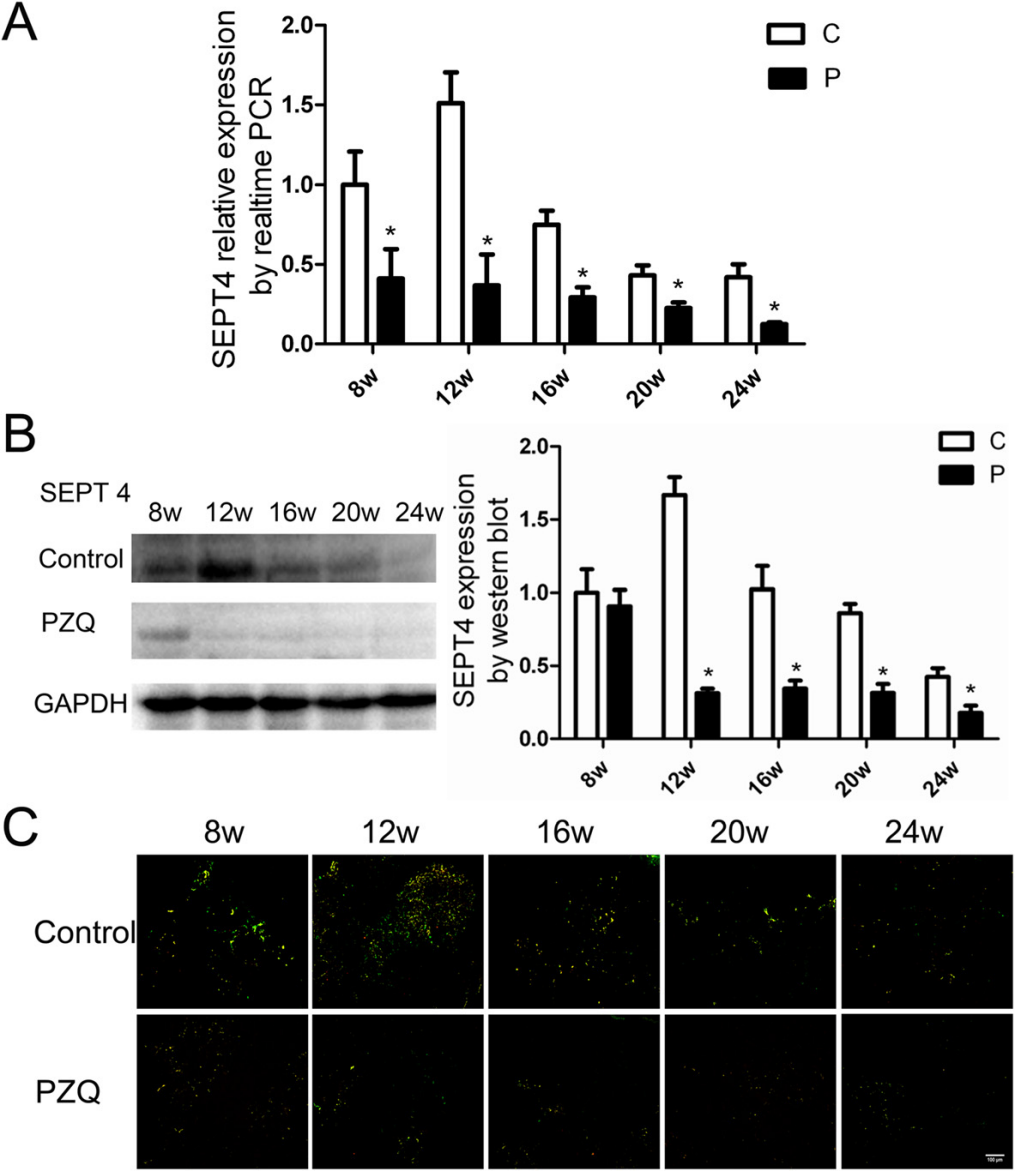
**Dynamics of SEPT4 expression in the livers of mice by western blot, qRT-PCR and immunofluorescence.** Anti-parasite treatment with PZQ reduced the expression of SEPT4 at 8 weeks in transcriptional level **(A)** and at 12 weeks in translational level **(B)**. **(C)** The co-localized cells (yellow) were significantly decreased after the treatment of PZQ compared with the infected group. Bar: 100 micrometer. * p < 0.05 vs the control group (without the treatment of PZQ).

Next, we detected the dynamic changes of SEPT4 by western blot and qRT-PCR. Firstly, we observed the changes in protein level. In the infected group, SEPT4 was found at a low level in normal liver tissue, and it increased progressively as the pathological progress. At 12 weeks, expression of SEPT4 peaked, then decreased gradually and returned to normal levels at 24 weeks. However, compared with the infected group, SEPT4 peaked at 8 weeks and had a significant decrease between 12 weeks and 24 weeks in PZQ treatment group (Figure 4A). The tendency of SEPT4 at the level of transcription was similar to protein level in the infected group, but it decreased promptly at 8 weeks after the treatment with PZQ (Figure 4B).

Then, we observed dynamics of SEPT4 co-localized with desmin-positive cells in areas of peri-sinusoidal space by double-immunofluorescence experiment. The number of desmin and SEPT4 double positive cells increased in the livers infected with *S. japonicum* for 12 weeks but decreased in the livers infected with *S. japonicum* for 24 weeks. After the treatment of PZQ, the double positive cells were decreased significantly at 12 weeks and stayed at a low level during the later time (Figure 4. C). These results show that SEPT4 is down-regulated at the translational and transcriptional level after the treatment of PZQ.

## Discussion

Hepatic schistosomiasis is a debilitating disease of human in which schistosome eggs become lodged in the host liver, leading to the formation of inflammatory granulomas and subsequent liver fibrosis. HSCs are the main sources of collagen in the liver and the main effector cells contributing to the formation of fibrosis. The aim of anti-fibrotic therapies are inhibiting the accumulation of HSCs and preventing the deposition of ECM [30].

SEPT4, expressed exclusively in quiescent HSCs in mouse and human liver [15,16], is down-regulated during myofibroblastic transformation of mouse HSCs *in vitro*, and that genetic loss of mouse Sept4 consistently augments liver fibrosis in CCl_4_, BDL and MCD diet models of liver diseases, indicating that SEPT4 is involved in suppressive regulation of myofibroblastic transformation and fibrogenesis in liver fibrosis [15]. In line with this, it is observed that loss of SEPT4 reduced the expression of Dkk2 that resulted in pro-fibrotic transformation of HSCs [17]. Based on these findings, it suggested that the up-regulation of SEPT4 probably had the ability to interfere with the process of transdifferentiation of HSCs. In our previous study, SEPT4 expression was up-regulated with the development of liver fibrosis and peaked at 12 weeks, then it was down-regulated from the chronic infection stage to the advanced stage [19]. The discrepancy may be due to the use of different models, which could differentially influence the expression of SEPT4. In vitro LPS enhanced the secretion of pro-inflammatory cytokines integral to the inflammatory response and leaded to the up-regulation of SEPT4 in HSCs through TLR4 and TGF-β pathway [18]. On the other hand, loss of SEPT4 reduced the up-regulation of the Dkk genes [17], which are implicated in the negative regulation of Wnt-mediated inflammation and pro-fibrotic reactions in the liver [31,32]. Thus, SEPT4 might be closely associated with the inflammatory response.

Inflammation is a common feature in many chronic liver diseases and closely related to the development of liver fibrosis [33,34]. Notably, HSCs are essential in the pro-inflammatory signaling pathways. It was reported that LPS elicited a variety of inflammatory responses and induced NO, IL-6 and TNF-α production in activated rat HSCs through p38/NF-κB signaling [35]. TNF-α is reported as acute-response cytokine [36,37] and mediate activation of NF-κB pathway in HSCs [38]. On the other hand, IL-6 directly promotes HSC survival and proliferation during enhanced liver fibrosis [39]. During the etiology of schistosome infection, the formation of multi-cellular granulomatous inflammation surrounding eggs is the classic phenomenon in the liver and intestines [40]. The inflammation initially recruited numerous inflammatory cells, such as eosinophils, macrophages and lymphocytes. Subsequently, the granulomatous inflammatory process induces HSCs to transform a quiescent phenotype to an activated state, proliferate and migrate to the peripheral regions of egg granulomas [25]. Our present study showed that pro-inflammatory cytokines IL-6 and TNF-α were all peaked at 8 weeks p.i.. Simultaneously, the expression of SEPT4 augmented with the peak at 8 weeks p.i.. Based on these findings, the increased expression of SEPT4 might be due to the inflammatory responses caused by *S. japonicum* egg.

PZQ is quite effective,safe,cheap and has little serious side effects against schistosomiasis [41]. It eliminates the adult worms and reduces eggs deposition, and it also has anti-granuloma formation and anti-inflammatory properties. The long-term administration of PZQ suppresses the formation of schistosome egg granulomas, including reduction in the areas of granulomas and the number of the fibroblasts within granulomas and the inflammatory cells [36,42]. Consistent with the results, we observed that the liver fibrosis was significantly reduced and the inflammation response was obviously alleviated by the down-regulation of pro-inflammatory cytokines IL-6 and TNF-α after the treatment with PZQ. Meanwhile, we also found that the expression of SEPT4 declined in the liver of PZQ treatment group. It indicated that anti-parasite treatment of PZQ significantly down-regulated the expression of SEPT4 on HSCs, possibly through the alleviation of the inflammatory responses.

## Conclusion

This study observed that the dynamic alteration of inflammatory response and SEPT4 during the *S. japonicum* egg-induced liver fibrosis after the administration of PZQ. The inhibitation of the expression of the SEPT4 by PZQ might be due to alleviation of the inflammatory response at the chronic and advanced stage of *S. japonicum* infection. Nevertheless, the mechanisms that the inflammatory responses influence the expression of SEPT4 should be investigated further. Therefore, in future research, we will investigate the molecular mechanism between inflammation and SEPT4.
